# Impact of artificial intelligence and digital technology-based diagnostic tools for communicable and non-communicable diseases in Africa

**DOI:** 10.4102/ajlm.v13i1.2516

**Published:** 2024-11-21

**Authors:** Chikwelu L. Obi, Joshua O. Olowoyo, Thembinkosi D. Malevu, Liziwe L. Mugivhisa, Taurai Hungwe, Modupe O. Ogunrombi, Nqobile M. Mkolo

**Affiliations:** 1 School of Science and Technology, Sefako Makgatho Health Sciences University, Pretoria, South Africa; 2 Department of Health Sciences and The Water School, Florida Gulf Coast University, Fort Myers, Florida, United States; 3 Department of Biology and Environmental Sciences, School of Science and Technology, Sefako Makgatho Health Sciences University, Pretoria, South Africa; 4 Department of Physics, School of Science and Technology, Sefako Makgatho Health Sciences University, Pretoria, South Africa; 5 Department of Physics, School of Physical and Chemical Sciences, North-West University, Mahikeng, South Africa; 6 Department of Computer Science and Information Technology, School of Science and Technology, Sefako Makgatho Health Sciences University, Pretoria, South Africa; 7 Department of Clinical Pharmacology and Therapeutics, School of Medicine, Sefako Makgatho Health Sciences University, Pretoria, South Africa

**Keywords:** artificial intelligence, diagnostic laboratories, communicable diseases, non-communicable diseases, machine learning, deep learning, Internet of Things

## Abstract

**Background:**

Artificial intelligence (AI) and digital technology, as advanced human-created tools, are influencing the healthcare sector.

**Aim:**

This review provides a comprehensive and structured exploration of the opportunities presented by AI and digital technology to laboratory diagnostics and management of communicable and non-communicable diseases in Africa.

**Methods:**

The study employed the Preferred Reporting Items for Systematic Reviews, Meta-Analyses guidelines and Bibliometric analysis as its methodological approach. Peer-reviewed publications from 2000 to 2024 were retrieved from PubMed^®^, Web of Science™ and Google Scholar databases.

**Results:**

The study incorporated a total of 1563 peer-reviewed scientific documents and, after filtration, 37 were utilised for systematic review. The findings revealed that AI and digital technology play a key role in patient management, quality assurance and laboratory operations, including healthcare decision-making, disease monitoring and prognosis. Metadata reflected the disproportionate research outputs distribution across Africa. In relation to non-communicable diseases, Egypt, South Africa, and Morocco lead in cardiovascular, diabetes and cancer research. Representing communicable diseases research, Algeria, Egypt, and South Africa were prominent in HIV/AIDS research. South Africa, Nigeria, Ghana, and Egypt lead in malaria and tuberculosis research.

**Conclusion:**

Facilitation of widespread adoption of AI and digital technology in laboratory diagnostics across Africa is critical for maximising patient benefits. It is recommended that governments in Africa allocate more funding for infrastructure and research on AI to serve as a catalyst for innovation.

**What this study adds:**

This review provides a comprehensive and context-specific analysis of AI’s application in African healthcare.

## Introduction

The Fourth Industrial Revolution epitomises the current state of emerging technologies, including how the Internet of Things, which includes devices that can communicate, sense, and exchange data with other devices and systems through the Internet, and artificial intelligence (AI) are influencing the ways we undertake different endeavours of life.^1^ It unravels the implications for skills development in different sectors, including healthcare. It also affords opportunities to reimagine and re-invent ways of teaching and learning in Africa. The Fourth Industrial Revolution includes digital technology and AI, gene sequencing, quantum computing, nanotechnology, and big data analytics, all of which are of significance in laboratory diagnostics.^2^ Big data involves large volume, high speed, and multiplicity of information processing for better insights, decision-making and automation.^2^ Quantum computing is useful in healthcare, including machine learning (ML) methods to diagnose diseases.^1^

Artificial intelligence is an emerging area in science and technology that may soon change every aspect of human life.^3^ Kayembe and Nel define AI as computer systems that can complete complex functions related to human intelligence.^1^ Artificial intelligence is the use of technologies that provide alternatives to human functions by allowing machines to perform effective functions that were previously carried out by humans by mimicking human action.^3^ From the foregoing, AI could thus be seen as a subset of digital transformation to perform different tasks that require human intelligence using computing devices such as robots.^4^ It is evident from the literature that AI alters the way humans perform their functions and leads to increased productivity in various sectors.^5^ Sub-fields of AI include: *Machine Learning*, which involves pattern discovery and evaluation from data sets, leading to machines’ performance improvement as the machines learn; *Deep Learning*, a sub-field of ML, comprising neural networks to facilitate ML in order for machines to formulate their own decisions; *Natural Language Processing*, a way that computers unearth data on human language to formulate decisions; and *Computer Vision*, which enables computers to acquire information and understanding from images or videos.^6^ Apart from what has been discussed earlier, AI technologies also encompass rule-based systems, robotic processing automation, clinical decision support systems, and generative adversarial networks, among others.^6^

Even though AI is a rapidly growing field all over the world, the adoption rate varies between developed and developing countries.^7^ In some countries, arguments against the use of AI include ethical use, which may violate the rights of citizens and cause loss of employment, especially for those with low skills.^8^ However, it has been estimated that AI will proliferate in many industries and contribute over 15.7 trillion United States dollars to the world economy by 2030.^9^ In 2023, business sectors in the United States, according to a report by Sahni and Carrus,^10^ are rapidly accepting the use of AI, except for the health sector, which has a low adoption rate of about 5%, even though AI is estimated to lead to 5% – 10% cost savings. In Europe, the Council of Europe’s AI Convention 2023–2024 used a human rights-based approach and reported that AI could be beneficial if formulated properly.^9^

In Africa, AI is currently infiltrating the African system through various technologies.^3,6,7^ In the early 1950s to 1970s, AI was based on machine development for inferences and decisions. During the ‘AI winter’ period (1970s to 2000s), funding was reduced, and fewer developments were achieved. From the late 2000s to 2020s, there was a seminal advancement in AI and digitalised medicine became more readily available; this was prompted by improved computer hardware and software programs.^6^ For example, through applications of deep learning in Kenya, healthcare services are now available to people without visiting hospitals or medical doctors, and in Nigeria, Zenvus, an electronic sensor, provides soil component information to farmers.^3,6^ South Africa has witnessed appreciable growth in AI research and application, especially in relation to power utilities and tax compliance.^11^ Also in South Africa, the Mukuru mobile phone application is assisting immigrants to send money home to their loved ones without physically visiting a bank for such transactions.^11^ In 2020, Okolo et al. also noted that the implementation of AI in African laboratories, particularly in low-resource contexts, is a growing area of interest.^12^ The 2023 report of Manson^13^ showed that AI has been included in some fields of medicine and laboratories in Africa. Manson also noted that AI can support knowledge-based treatment planning in the field of radiotherapy, although prior knowledge and inclusion in curricula for training and education may be important.^13^

The application of meaningful medical AI has been shown to occur more in developed nations in comparison to what is found in Africa.^8^ To address this, the United Nations has indicated that there is a need for all stakeholders to be brought together to deliberate on how AI can be used for the provision of critical services in the public sector so that the sustainable development goals can be achieved.^8^ The introduction of AI in World Health Organization African regions is still emerging and there is a lack of studies or research undertaken due to inadequate resources, lack of infrastructure, and lack of knowledge about AI among healthcare practitioners compared to those in better-resourced, high-income countries.^14^

The use of information and communications technology through management systems employing mobile tools and technologies has become the most relevant emerging trend in the monitoring of non-communicable diseases in Africa due to their portability, low cost, continuous connectivity, personalised effects, and ease of use.^8^ Nevertheless, there has been limited literature on the use of key enabling technologies and AI in sub-Saharan African countries and there is still a need for further studies to show whether AI may still be crucial in the expansion of aids for diagnosis or advancing signals from the use of affordable sensors which can be produced easily.^15^

Within the healthcare sector in Africa, the use of AI is growing, though at a lower speed when compared to other regions of the world.^16^ In African countries, the dissemination of information on health and other areas is done via the news media, involving television and radio services using modern technology such as cell phones or other media.^8,16^ Despite this slow development in some countries of sub-Saharan Africa, AI has been used in some instances to carry out various duties, which include the prediction of diseases and outbreaks, determining the extent of a disease, performing homecare and distance monitoring of patients in some settings.^17^ In Nigeria, Mali, Senegal, and Burkina Faso, among others, scientists have developed models to monitor and predict the spread of malaria using ML.^18^ In South Africa and Zambia, according to a 2021 report by Yadav,^19^ AI has been used to monitor and survey the epidemiology of tuberculosis, and HIV AIDS. In 2020, Onu et al. also reported that in Nigeria, a program called ‘Ubenwa’ used AI to improve the diagnosis of birth asphyxia in low-income and rural areas of the country.^20^

One of the greatest challenges for most African countries is the current episode of brain drain, which is not limited to the health sector only. Most countries in Africa lose qualified healthcare personnel to wealthier countries, both within and outside the region. The use of AI in the African health sector may thus assist in filling these gaps by providing improved diagnosis, treatment, and disease monitoring programmes, especially in rural areas.^21^

However, there are several drawbacks in the adoption of AI in developing countries, which may include data availability, analysis, and infrastructure.^22^ In addition, the state of laboratories and hospitals, level of education, shortage of skills, and development of appropriate software may impact the application of AI, thus leading to an increased burden of communicable and non-communicable diseases.^21,22^ Non-communicable diseases are diseases that are typically chronic and not directly transmitted from one person to another; they include cardiovascular disease, diabetes, and cancer.^23^ Communicable diseases are infectious diseases which spread by contact with contaminated surfaces, bodily fluids, blood products, insect bites, or through the air.^23,24^ In Africa, 31.4 million people die annually from non-communicable diseases, including cancer, diabetes and cardiovascular disease, with 71% of deaths in the 30–70 years age range.^24^ The three most prevalent communicable diseases in Africa are HIV/AIDS, malaria and tuberculosis, and are responsible for nearly 80% of the total infectious disease burden, claiming more than 6 million people per year in Africa.^23,24^ According to Oronti,^25^ recently in 2024, there has been a need for the world to move away from responses to the coronavirus disease 2019 pandemic and to address the other communicable and non-communicable diseases which account for most of the expenses associated with healthcare and mortality. The United Nations General Assembly has proposed the Sustainable Development Goal 3.4 target to decrease by one-third the untimely deaths from communicable and non-communicable diseases which have become a global major challenge in the past decade.^26^ By 2030, investments in digital health technologies, such as telemedicine, devices that can be worn, and AI, should be used to offer support to accomplish the United Nations Sustainable Development Goal for health in Africa.^26^

One of the key aspects of this review is to highlight the current state, potential and challenges of AI and digital technology in African laboratories and hospitals, especially in the diagnosis and treatment of communicable and non-communicable diseases in the past 24 years. An overview of the healthcare system is germane to the subject matter.

### Significance of the review

The novelty of this review lies in its comprehensive and context-specific analysis of AI’s application in African healthcare. It provides a holistic overview of current technological implementations and potential benefits, with a particular focus on the unique infrastructural and socio-economic conditions of African countries. Moreover, it also provides the current state of AI and digital technology, and their impact in healthcare and laboratory diagnostics of communicable and non-communicable diseases in Africa. By addressing ethical considerations such as equitable access, the review underscores the necessity of robust regulatory frameworks. Additionally, it offers practical, actionable recommendations for governments, educational institutions, and healthcare organisations, promoting targeted investments and initiatives to enhance AI adoption. The review also highlights significant gaps in research, setting an agenda for future efforts and encouraging collaborative research between African and international partners. This focus on reducing disparities and fostering digital inclusion aims to ensure sustainable health improvements and drive innovation in AI applications for healthcare across the continent.

### Aim and objectives

The aim is to provide a comprehensive and structured exploration of the opportunities presented by AI and digital technology to laboratory diagnostics and the management of communicable and non-communicable diseases in Africa.

To achieve this aim, the study will systematically identify, narrate and analyse the various opportunities of AI and digital technology in healthcare and laboratory diagnostics in Africa. Additionally, it will evaluate the current state of AI and digital technology in healthcare and laboratory diagnostics in Africa, identify challenges, and make recommendations.

## Methods

The Preferred Reporting Items for Systematic Reviews and Meta-Analyses standards and bibliometric analysis were employed in the study.^27^ This was to ensure transparency, consistency and controlled or structured reproducible review processes. Inclusion and exclusion criteria, and a thorough search strategy, formed part of the methods. Qualitative thematic analysis was employed for a comprehensive literature assessment on AI’s prospects for laboratory diagnostics of communicable and non-communicable diseases, including narratives on salient themes, concepts, or patterns among the chosen studies.

### Retrieval of evidence and data analysis

The researchers utilised databases of PubMed^®^ (United States National Library of Medicine, Bethesda, Maryland, United States [https://pubmed.ncbi.nlm.nih.gov], Web of Science™ Core Collection Database (Clarivate Analysis, Boston, Massachusetts, United States [https://clarivate.com/products/scientific-and-academic-research/research-discovery-and-workflow-solutions/webofscience-platform/web-of-science-core-collection]) and Google Scholar (https://scholar.google.com) in July 2024, to find peer-reviewed articles that had been written about AI and digital technology in African healthcare. Different kinds of research articles and study designs were included in this search. However, the selection of the communicable and non-communicable diseases was based on prevalence in Africa. As mentioned in the foregoing section, the three most prevalent communicable diseases in Africa are HIV/AIDS, malaria and tuberculosis.^23,24^ We also based our selection on non-communicable diseases such as cancer, cardiovascular disease and diabetes, which are the most prevalent non-infectious diseases in Africa.^23,24^ The designated diseases were integrated interchangeably with the keywords ‘artificial intelligence’ OR ‘deep learn’ OR ‘machine learning’ OR ‘neural network’ OR ‘compu Intelligent’ OR ‘robot’ for assessing the impact of AI and digital technology on communicable and non-communicable diseases in Africa. We limited the publication years from 2000 to 2024. We excluded non-English language articles that were not focused on healthcare in Africa.

Extracted data were synthesised thematically to identify patterns, trends, and key insights related to the opportunities presented by AI and digital technology-based diagnostic tools for communicable and non-communicable diseases in Africa. Clusters that reflected commonalities and areas of focus were identified. Emerging trends and patterns within the selected literature were noted. Moreover, GraphPad Prism v. 10.2.3 (GraphPad, La Jolla, California, United States [https://www.graphpad.com/features]) was utilised for statistical analyses. The Pearson correlation was calculated to identify disproportionate national scientific and socio-economic characteristics, association between publication output and gross domestic expenditures for research and development (GERD), number of research funding agents, and gross domestic product (GDP). Variables were expressed as percentage and mean ± standard deviation.

## Results

### Current state and opportunities of artificial intelligence and digital technology

**FIGURE 1:**
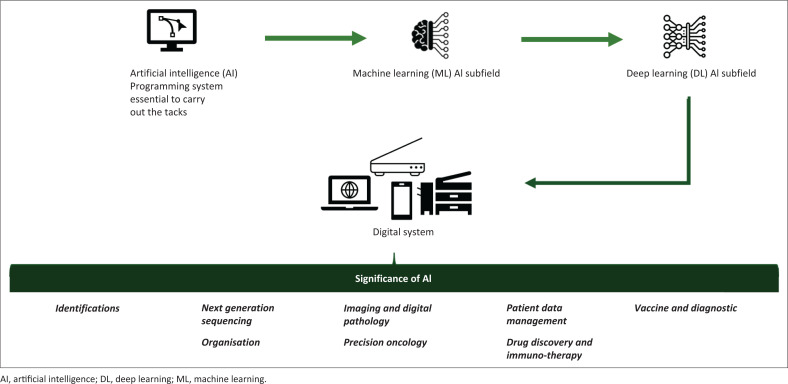
Illustration of artificial intelligence and digital technology, and their importance in an African laboratory environment. Artificial intelligence has several applications in the sector of digital healthcare.

Comprehending the present status of AI and digital technology research in the African healthcare sector is crucial for creating AI solutions that are customised to the unique circumstances found on the continent, such as the high incidence of infectious diseases and resource scarcity. With the integration of AI and digital technologies, the evidence from the perused PubMed^®^, Web of Science™ and Google Scholar databases suggests that the African healthcare landscape is undergoing an early-stage revolutionary shift. These improvements are most noticeable in laboratory settings, where AI is helping to improve tailored treatment, surveillance for communicable and non-communicable diseases, and diagnostic competencies. Figure 1 displays the medical uses of AI domains and subdomains in Africa.

**FIGURE 2:**
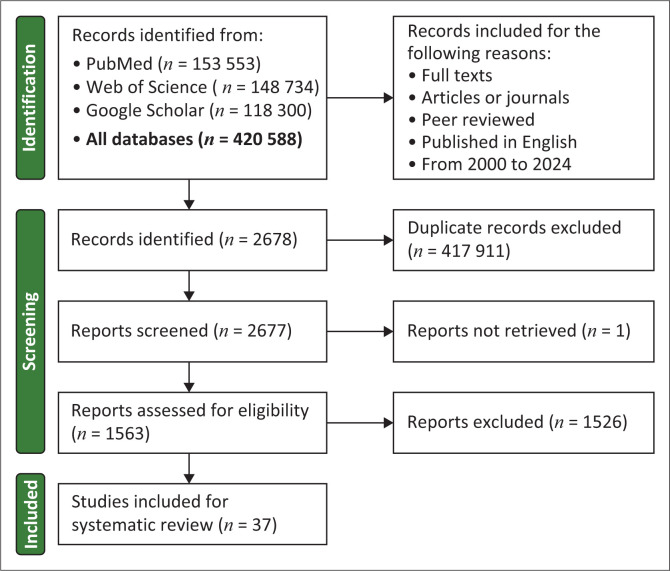
Preferred Reporting Items for Systematic Reviews and Meta-Analyses flow diagram for evaluating the impact of artificial intelligence and digital technology-based diagnostic tools for communicable and non-communicable diseases in Africa.

**FIGURE 3:**
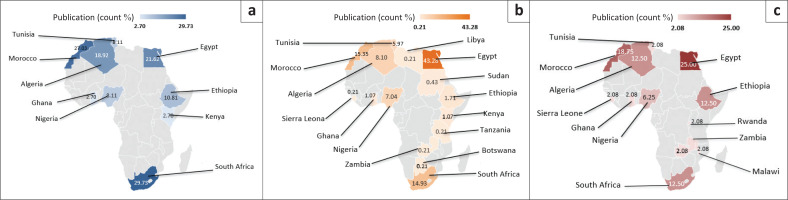
Overall research status performance of artificial intelligence and digital technology in healthcare and laboratory diagnostics of non-communicable diseases in different African countries: (a) cardiovascular disease, (b) cancer and (c) diabetes.

The study incorporated a total of 1563 peer-reviewed scientific documents focusing on research of AI and digital healthcare for communicable and non-communicable diseases that are most prevalent in Africa; after filtration, only 37 were utilised for systematic review (Figure 2). Table 1 depicts various opportunities of AI and digital technology in healthcare and laboratory diagnostics in Africa. In reference to non-communicable diseases (Figure 3), the three most dominant African countries with high AI and digital technology related to cardiovascular disease research productivity are South Africa (29.73%), Morocco (27.02%), and Egypt (21.62%). In the case of the research field of AI and digital technology related to diabetes and cancer diseases, the same three countries remained dominant. Egypt (25.00% for diabetes; 43.28% for cancer), was ranked as a high-producing African country in these research fields of AI and digital technology related to diabetes and cancer diseases. Morocco (18.75% for diabetes; 15.35% for cancer) ranked second, and South Africa (12.50% for diabetes; 14.92% for cancer) ranked third.

**TABLE 1:** Classification of various opportunities of artificial intelligence and digital technology in African healthcare and laboratory diagnostics.

Disease type	African countries	AI method	Digital technologies (IoT applications)	Applications	References
**Non-communicable diseases**					
**Cardiovascular Disease**					
	South Africa Morocco Egypt Algeria Ethiopia Nigeria Tunisia Kenya Ghana	Deep learning Machine learning Convolutional neural network	IoT communication technologies Wearable devices Sensors e.g. electrocardiogram Telecommunication tools	Patient data management, mobile phones and SMS interventions. Telemedicine for remote monitoring and consultation management. Disease prediction, AI for early detection and prediction of heart diseases through analysis of medical records and wearable devices data. Wearable devices for continuous monitoring of blood pressure.	^28,29,30,31,32,33,34,35,36,37^
**Diabetes**					
	Egypt Morocco South Africa Algeria Ethiopia Nigeria Tunisia Ghana Sierra Leone Rwanda Zambia Malawi	Deep learning Machine learning Convolutional neural network Artificial neural network	Biosensors e.g. Continuous Glucose Monitoring and smart bands IoT communication technologies Wearable devices, e.g. body area network	AI algorithms for personalised treatment plans and monitoring, and mobile apps for blood sugar tracking and management.	^38,39,40,41,42,43,44,45^
**Cancer**					
	Egypt Morocco South Africa Algeria Nigeria Tunisia Ethiopia Ghana Kenya Sudan Botswana Libya Sierra Leone Tanzania Zambia	Deep learning Machine learning Convolutional neural network Deep neural network	IoT communication technologies Sensors Mammograms Magnetic resonance imagery	AI in diagnostics through image recognition. Predictive analytics for identifying high-risk patients. Tele-oncology for remote consultation and treatment follow-up.	^46,47,48,49,50,51,52,53,54^
**Communicable diseases**					
**HIV/AIDS**					
	South Africa Nigeria Ethiopia Kenya Cameroon Ghana Zimbabwe Botswana Tanzania Uganda	Deep learning Machine learning Convolutional neural network	IoT communication technologies Wearable devices Telecommunication tools	Used for predicting outbreak patterns and optimising treatment plans. Mobile health apps for medication adherence and patient education.	^55,56,57,58^
**Tuberculosis**					
	South Africa Nigeria Morocco Egypt Tunisia Uganda Algeria Kenya Zimbabwe Cameroon Ghana Sudan Rep Congo	Deep learning Machine learning Convolutional neural network	IoT communication technologies; Wearable devices; and telecommunication tools	Used for rapid diagnostic tests and predictive analytics for treatment outcomes. Mobile health apps for monitoring treatment adherence.	^59,60,61,62,63^
**Malaria**					
	South Africa Nigeria Ghana Egypt Kenya Morocco Ethiopia Uganda Sudan Tunisia Botswana Tanzania Algeria Cote Ivoire Gambia Rwanda	Deep learning Machine learning Convolutional neural network	IoT communication technologies; Wearable devices; and telecommunication tools	Used for predicting malaria outbreaks based on environmental and climatic data. Mobile apps for reporting and tracking malaria cases.	^64,65,66,67^

Note: Please see the full reference list of this article for details on the articles cited: Obi CL, Olowoyo JO, Malevu TD, et al. Impact of artificial intelligence and digital technology-based diagnostic tools for communicable and non-communicable diseases in Africa. Afr J Lab Med. 2024;13(1), a2516. https://doi.org/10.4102/ajlm.v13i1.2516.

AI, artificial intelligence; apps, applications; IoT, Internet of Things; SMS, short message service.

**FIGURE 4:**
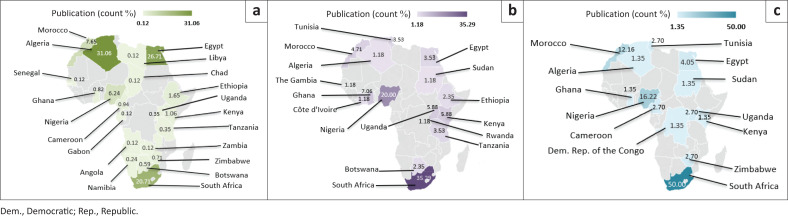
Overall research status performance of artificial intelligence and digital technology in healthcare and laboratory diagnostics of communicable diseases in different African countries: (a) HIV/AIDS, (b) malaria and (c) tuberculosis.

Regarding communicable diseases (Figure 4), the countries that dominate in the research field of AI and digital technology related to HIV/AIDS diseases are Algeria (31.06%, ranked first), Egypt (26.71%, ranked second), and South Africa (20.71%, ranked third). However, South Africa was ranked as the highest-producing African country in the research field of AI and digital technology related to tuberculosis (50.00%) and malaria (35.29%), and Nigeria ranked second (tuberculosis, 16.21%, and malaria, 20%). Morocco ranked third in the fields of AI and digital technology related to tuberculosis (12.16%), and Ghana ranked third in the fields related to malaria (7.05%).

### Artificial intelligence and digital technology for non-communicable diseases in Africa

#### Cardiovascular disease

One of the digital technologies that is common in sub-Saharan countries is the mobile phone, which is used by almost 45% of the population.^28^ The technology of mobile phones, in particular the use of phone calls, mobile applications and short message service (SMS), has been the most reported use of a variety of technology applications for dealing with hypertension in Africa.^28^ Even though there has been more interest in the use of mobile phones for healthcare purposes in children, maternal health and infectious diseases in sub-Saharan countries, there has been an escalating interest in the use of mobile phones in non-communicable diseases.

According to a study by Stokes et al. in 2022, the strategy of using mobile communications technology in sub-Saharan countries and Africa as a whole has been shown to have a positive impact on the control of hypertension that is related to cardiovascular diseases through adherence to treatment and health knowledge.^29^ In addition, there was an indication of positive perceptions by the stakeholders on the management and prevention of hypertension. In Cape Town, South Africa, an intervention using an automated system with either interactive or informational SMS text messages was used for remote delivery of reminders for pick-up of medication, support for medication adherence, education related to cardiovascular diseases, and clinic appointments.^30^ In this study, a low-cost system was based on a commonly available mobile technology to deliver the SMS-based intervention for the management of communication with the patients.^30^

The SMS system, together with voice mail reminder messages, was found to be more applicable to health education, adherence to taking medication, healthy diets, and promotion of physical exercises than to early prognosis, diagnosis, and detection.^31^ A school of thought believes that the general application of the SMS intervention was found to be doubtful and inapplicable to every sector of the African population due to low literacy levels that are still prevalent in many parts of Africa.^15^ Hence, there was a need to reassess or further engage these innovative ways of providing new focus or other combinations with the SMS. The use of SMS was also shown to enhance the positive behaviour of patients, even though this was not shown to have contributed to reductions or improvements in the control of hypertension, which may result in an enlarged heart.^15^

However, the use of techniques such as ML and AI have been considered to offer superior and innovative ways for providing efficient and low-cost ways of diagnosing, monitoring and managing non-communicable diseases related to hypertension and cardiovascular diseases.^28,32^ It was hence recommended that other avenues that could be employed to combat high literacy and lack of specific knowledge were applications that use graphical illustrations and pictures and prudent design principles. In addition, ML, three-dimensional printing technologies and AI could be employed successfully through elaborate research that was based on quality and evidence-based results in the health sector in Africa.^28,32,33,34^

One of the mHealth tools that has been used in Africa for intervention to monitor blood pressure in Ghana, included the use of a Bluetooth-enabled blood pressure device together with a smartphone application to monitor the readings of blood pressure and the intake of medications under the guidance of a nurse.^31^ In this study, there was a positive attitude and satisfaction with the intervention by the majority of the participants who had suffered a stroke, with some of the participants even stating that they regretted not having been exposed to the intervention earlier. The technologies in mHealth have the potential to achieve a greater implementation to assuage the burden of non-communicable diseases in Africa.^28^ According to Opoku et al. in 2017, the impact of mHealth interventions in sub-Saharan African countries to improve healthcare and treatment through access to specialised services which were previously unavailable in a remote manner, had become increasingly beneficial.^35^ According to Stephani^36^ in 2016, it is not possible as yet to conclude on the efficiency of mHealth interventions due to a limited number of studies and vast variations in the reported outcomes and heterogeneity of the mHealth interventions evaluated. Hence, there is a need for further research to understand the particular effects of different types of mHealth interventions on a variety of people with non-communicable diseases in low- and middle-income countries.^36,37^

#### Diabetes

In Rwanda, screening that was supported by AI increased the opportunity for the provision of immediate counselling and health education on eye care for diabetic patients who required referral.^38^ These referrals contributed to enhanced adherence in grading of diabetic retinopathy when compared to cases where there was delayed communication of results that were done and graded by healthcare workers.^38,39^ As a result, AI screening was shown to provide a crucial benefit in promoting adherence to the recommended management of diabetic eye care in sub-Saharan Africa.^40,41,42,43^ A similar study on the importance of AI diabetic retinopathy screening software in combination with a cheap hand-held fundus camera, to cater for the needs of patients with diabetes in a hospital, was carried out in Malawi.^44^ Results of the study showed that the AI software was able to pickup images that did not satisfy the quality standards for the precise prediction of diabetic retinopathy grading.^44^

The AI model using deep learning showed comparable outcomes with humans in the detection of prevalence of referable diabetic retinopathy and associated risk factors in the screening programme of a population in Zambia.^45^ The AI model was shown to produce clinically suitable performance in the detection of diabetic retinopathy that could be referred.^45^

#### Cancer

The application of AI in the development of reliable tools for cancer outcome predictions in Africa is a fairly new attempt.^46^ Nevertheless, the advancement and endorsement of the technical aspects of AI models related to cancer in Africa are on the whole satisfactory; this may be due to the available knowledge of people with adequate skills to undertake ideal model selection, pre-processing of data, validation, tuning of data, and deployment of data.^47,48,49,50,51,52,53^ However, the models that have been developed have not yet been evaluated for their efficiency and impact in assistive automated clinical risk stratification and decision-making. There is an expectation that there will be an integration of digital health technologies and AI-based predictions to alleviate the challenges of diminishing facilities, manpower, and lack of access to healthcare that are being experienced in the management and diagnosis of cancer in Africa.^45^

The third most common cancer contributing to death is colorectal cancer in sub-Saharan Africa, with South Africa having the highest incidence.^53^ Supervised ML algorithms used together with statistical algorithms have been shown to be able to offer crucial predictive factors for recurrences of colorectal cancer, survival of the patients, and enhanced interpretation of colorectal cancer globally. The importance of ML is that it can be used for the extrapolation of the data that are collected externally from the hospitals, because the variables that regulate the outcome are as important as the pre-hospital detection of colorectal cancer patients and the treatment prescribed by clinicians.^53^

In 2022, a study by Joseph et al. showed that the use of a handcrafted approach for the use of algorithms, such as deep neural network classifiers and feature extractors, enhanced performance in multi-classification of breast cancer in Nigeria, and that data augmentation played the main role in improving the accuracy of the classification of breast cancer using histopathological images.^54^

### Artificial intelligence and digital technology for communicable diseases in Africa

#### HIV/AIDS

In 2010, Singh and Mars investigated ML application for the prediction of future CD4 cell count change in South Africa, since CD4 cell count is a preferred surrogate marker and assists clinicians and other health practitioners with the management of the infection as well as with the allocation of the resources needed.^55^ It was reported that close monitoring of the progression of HIV infection by counting CD4 cells is vital to its effective management. They built a support vector machine classification model that predicted the degree of CD4 cell count change.^55^ The model took as input the genome, current viral load, and number of weeks from baseline CD4 cell count, and predicted the range of CD4 cell count change. The model produced an accuracy of 83%. This pilot project shows that a change in CD4 cell count may be accurately predicted using ML on genotype, viral load, and time. Moreover, there are several mobile health interventions for people living with HIV: cell phone-delivered reminders and inspirational messages to boost clinic attendance and antiretroviral therapy adherence, laboratory test delivery, and behaviour modification messages are some of the most common.^56,57,58^

#### Tuberculosis

Machine learning is often used together with signal processing methods to automate communicable disease diagnoses. Most interventions for diagnoses with the use of AI in low- and middle-income countries reported high specificity, sensitivity and accuracy to comparator diagnostic tools. Machine learning assists medical practitioners in diagnosing tuberculosis and malaria with expert systems.^59^ In 2020, Peiffer-Smadja^60^ reported the impact of current AI tools over a range of clinical outcomes such as the diagnosis of tuberculosis or surgical site infections, indicating sensitivity and specificity. The AI tool predicted a bacterial infection in individuals who were not identified by clinicians as having an infection on admission, but were diagnosed later with a bacterial infection.^60^ Moreover, AI and digital technologies are currently being used for drug discovery for several research studies.^61,62,63^

#### Malaria

In 2023, Silka et al. presented a novel convolutional neural network architecture for the detection of malaria from blood samples with a 99.68% accuracy.^64^ The novel convolutional neural network accurately classified infected and uninfected samples with high specificity and sensitivity. An analysis of model performance on different subtypes of malaria was performed and the implications of the findings for the use of deep learning in infectious disease diagnosis was determined.^16,17,64^ Moreover, there are several studies indicating the benefits of in silico research, including capacity to screen a huge number of drug candidates in a comparatively short length of time, which saves time and money when compared to traditional drug development methods.^65,66,67^

### Identification of national scientific and socio-economic characteristics challenges

#### Non-communicable diseases in Africa

**FIGURE 5:**
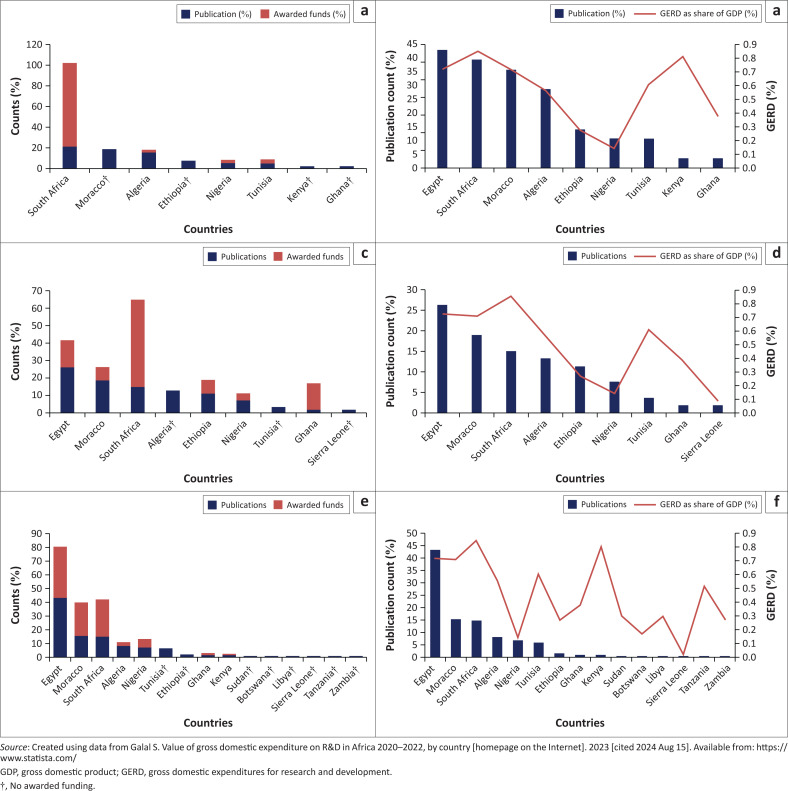
Correlational data of non-communicable disease research in Africa. Trends of research publications output, awarded funds for (a) cardiovascular disease, (c) diabetes and (d) cancer, and gross domestic expenditures for research and development as a share of gross domestic product for (b) cardiovascular disease, (d) diabetes and (f) cancer.

A Pearson correlation was performed to determine if there is a correlation between variable of publication outputs and variable of awarded funding or with GERD as a share of GDP (Figure 5). Cardiovascular disease research metric data exhibited a positive, but not significant, correlation (*r* = 0.5, *p* = 0.17) between variables of publication outputs (11.11 ± 8.25) and awarded funds (11.11 ± 26.26) even with the GERD as a share of GDP variable (0.56 ± 0.25; *r* = 0.5, *p* = 0.158). Egypt and South Africa are among the countries that are awarded more funding. Morocco, Ethiopia, Kenya, and Ghana did not declare their awarded funding (Figure 5a, b).

However, there was no significant positive correlation when metric data of diabetic disease research were utilised (*r* = 0.32, *p* = 0.394), between variables of publication outputs (11.11 ± 8.3) and awarded funds (11.11 ± 15.79). Similar findings were achieved, where variables of GERD as a share of GDP (0.56 ± 0.25) exhibited association with publication outputs (*r* = 0.35, *p* = 0.359). Although South Africa was awarded more funding (50%) compared to other African countries, the country had fewer publication outputs compared to Egypt and Morocco which were awarded less funding of 15.38% (Egypt) and 7.69% (Morocco). Algeria, Tunisia, and Sierra Leone did not declare their awarded funding (Figure 5c, d).

Cancer disease research metric data exhibited a significant correlation (*r* = 0.92, *p* = 0.001) between publication outputs (6.67 ± 11.41) and awarded funds (6.67 ± 12.22). There was also a significant positive association (*r* = 0.53, *p* = 0.044) between publication and GERD as a share of GDP (0.44 ± 0.26). Egypt, Morocco, and South Africa are among the top three countries with high research output productivity and more awarded funding. Tunisia, Ethiopia, Sudan, Botswana, Libya, Sierra Leone, Tanzania, and Zambia did not declare their awarded funding (Figure 5e, f).

#### Communicable diseases in Africa

**FIGURE 6:**
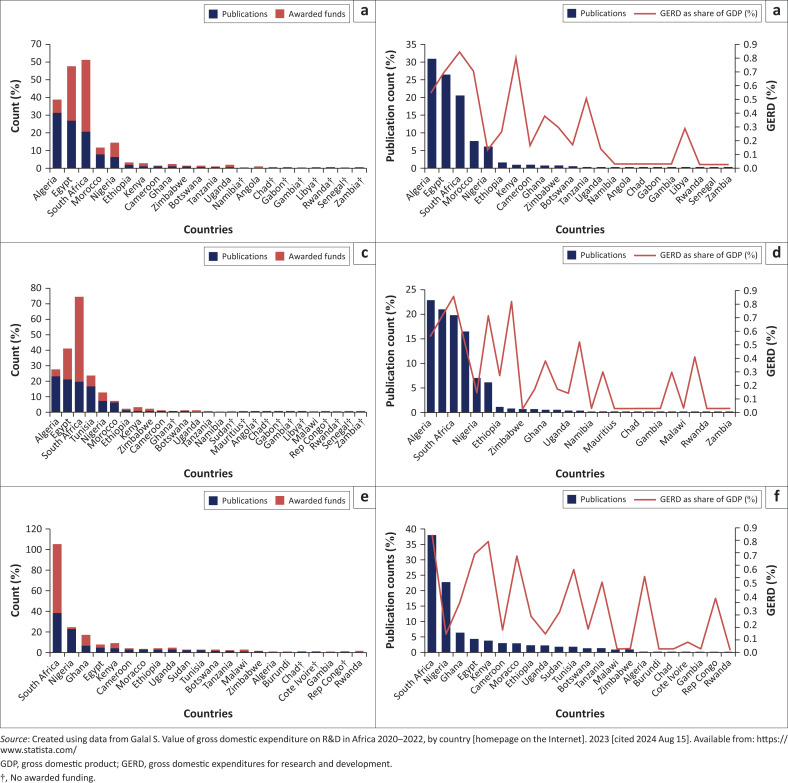
Correlational data of communicable diseases research in Africa. Trends of research publications output, awarded funds for (a) HIV/AIDS, (c) tuberculosis and (e) Malaria, and gross domestic expenditures for research and development as a share of gross domestic product for (b) HIV/AIDS, (d) tuberculosis and (f) Malaria.

Pearson correlation showed that there was a significant correlation (*r* = 0.79, *p* = 0.001) between publication outputs (4.55 ± 9.14) and awarded funds (4.55 ± 10.36), when metric data of HIV/AIDS research was utilised. Moreover, there was a significant positive association (*r* = 0.63, *p* = 0.002) between variables of publication outputs and GERD as a share of GDP (0.29 ± 0.28). Egypt (30.49%) and South Africa (40.32%) were awarded a greater amount of funding compared to other African countries. However, these countries had fewer publication outputs compared to Algeria, which was awarded less funding of 7.54%. Namibia did not declare its awarded funding (Figure 6a, b).

Metric data for tuberculosis research also exhibited a significant correlation (*r* = 0.69, *p* = 0.001) between publication outputs (3.70 ± 7.20) and awarded funds (3.70 ± 10.89). Moreover, there is no significant positive association (*r* = 0.365, *p* = 0.258) between publication and GERD as a share of GDP (0.27 ± 0.27). Even though South Africa received more funding, of approximately 54.25% compared to other African countries, it had fewer publication outputs compared to Algeria and Egypt which were granted less funding of 4.91% (Algeria) and 19.85% (Egypt). Ghana managed to publish research outputs despite the non-declared awarded funding (Figure 6c, d).

Metric data for malaria research also exhibited a significant positive correlation (*r* = 0.86, *p* = 0.001) between variables of publication outputs (4.55 ± 8.85) and awarded funds (4.55 ± 14.16). However, there was no positive association (*r* = 0.35, *p* = 0.359) between variables of publication outputs and GERD as a share of GDP variable (0.32 ± 0.28). South Africa had more publication outputs and greater funding compared to other African countries. Chad, Côte d’Ivoire and Democratic Republic of the Congo did not declare any awarded funding (Figure 6e, f).

## Discussion

The application of AI is changing the complex nature of healthcare due to its role in revolutionising healthcare systems, thereby enhancing healthcare delivery and patient satisfaction.^68^ Researchers have noted that the use of AI in the African health sector is still at the infant stage, which may necessitate a continuous monitoring process to identify potential risks and hazards associated with the use of AI.^69,70^ This view can be consolidated with our findings, where the research outputs evidence suggests that the African healthcare landscape is undergoing an early-stage revolutionary shift in terms of integrating AI and digital technologies. The application of AI and digital technologies can improve treatment effectiveness and efficiency through various data and identification processes.^68,71,72^ Since AI is a technology that relies on the reduction of human involvement in healthcare delivery, speeding up the process of summarising data and assisting in identifying common factors in a problem.^73,74,75^ Artificial intelligence may reduce the over-reliance on manpower, especially in countries where there are shortages of both skilled and unskilled workforce.^72^ From the patient’s perspective, AI can assist with data collection and management. This will ease the burden of repeated hospital visits on the part of patients and may also provide warning on issues of drug overdose, drug-to-drug interactions, and any unforeseen hazards.^73,76,77,78^

There are, however, some risks associated with the use of AI.^79,80,81,82,83^ A major consideration in the use of AI in laboratory diagnostics and healthcare in general are inequality or poverty, and income distribution in Africa.^84,85^ Our results reflected the disproportionate research outputs distribution across Africa for three most prevalent communicable and non-communicable diseases in Africa. In terms of non-communicable diseases research, South Africa, Egypt, and Morocco contribute 78.38% of the entire continent’s total cardiovascular disease research, 60% for diabetes research, and 73.56% cancer research, while for the communicable diseases research field, Algeria, South Africa, Nigeria, and Morocco contribute 86.11% towards the field of HIV/AIDS research, and 79.72% towards tuberculosis research. South Africa, Nigeria, and Ghana contribute 62.35% for the total research outputs in the malaria research field. These disproportionate research outputs can be elucidated by a several factors, including the reality that both South Africa and Egypt are rated as the top two countries with the highest GDP on the continent.^86^ Besides, South Africa, Morocco, and Egypt are still statistically recognised as the three African countries with the highest GERD, which is approximately between 6.2 and 8.86 billion United States dollars.^87^ Although, Nigeria and Ghana are rated in the top 16 African countries in terms of GERD, the results revealed that Nigeria contributes 54.25% towards the communicable diseases research field and Ghana contributes 21.82% for the non-communicable diseases research field.

There is a notion that only rich people will be able to afford new technologies for healthcare, while the poor may trail behind, eventually creating social unrest. As a matter of fact, the overall success of the use of AI to foster healthcare, including laboratory diagnostics, should be viewed in terms of the impact on people at various socio-economic levels, and their intercultural perspectives.^84^ Other challenges regarding the use of new technologies include inadequate funding for healthcare, exposure of healthcare workers to changing technologies, fostering self-learning and discovery, promoting imaginative and critical thinking, and systemic change management. The analysed data of this study depict special consideration to a clear correlating trajectory of Africa’s research outputs and obtained research funds. The designation of a superior share of South African local funding may possibly be rationalised given that in the year 2003, the South African government assigned a new funding system that is grounded in the research output of tertiary institutions.^87^ Moreover, South Africa spends approximately 1% of its GDP on research and development, as recommended by the African Union.^87,88^

Accordingly, the mapping of AI and digital health research in Africa can play an important role in providing strategic guidance to governments. This can assist the governments to prioritise investments in digital health technology according to the particularities of each country in establishing sustainable economic development and health returns.^84,85^ Hence, digital infrastructure should be in place to sustain the health sector and economy in most African countries.

### Recommendations

Based on the results of this review, the following recommendations are made to guide future AI implementation in laboratory diagnostics in Africa. To facilitate the widespread adoption of AI and digital technology in laboratory diagnostics across Africa, a multifaceted approach is recommended. Firstly, streamlining of the curriculum of universities in Africa, especially in medical schools, to align with the era of AI, digital technology, and the changing landscape of innovations and rapidly emerging technologies. This educational reform will empower future healthcare professionals with the requisite skills to effectively utilise these technologies in diagnostics. Healthcare practitioners should be taught the need to adapt readily to emerging situations by learning, unlearning and re-learning in order to catch up with the momentum of developments in the field. Secondly, concerted public awareness campaigns are essential to educate communities about the benefits and applications of AI in laboratory diagnostics, dispelling misconceptions and fostering trust. Policy formulators and decision-makers, including executive and legislative arms of governments in Africa, should make substantial improvements in healthcare delivery systems and infrastructural facilities to support AI laboratory diagnostics. Thirdly, substantial investments in infrastructure are imperative to support AI implementation, addressing challenges such as power outages and inadequate Internet connectivity. Additionally, comprehensive capacity-building programmes should be established to enhance the proficiency of healthcare professionals in utilising AI and digital tools for diagnostics, potentially through collaborations with international organisations and technology firms. Fourthly, African academic and research institutes and academics must develop self-governing sources of funding for federal and philanthropic gestures. This will enable Africans to introduce their special research agenda which will permit less reliance on international research funding agents. Fifthly, African countries must utilise a full 1% of GDP for GERD. Lastly, fostering collaborative research endeavours among researchers, healthcare practitioners, and technology experts is crucial for evaluating the effectiveness and feasibility of AI applications within the African context, thus informing evidence-based policies and strategies for scaling up AI adoption in healthcare. By implementing these recommendations, Africa can overcome existing barriers and realise the full potential of AI in improving laboratory diagnostics and enhancing healthcare delivery across the continent.

### Conclusion

It is concluded that while AI and digital technology are useful in the laboratory diagnosis of communicable and non-communicable diseases in Africa, the paucity of data and practices on such use shows that the method of diagnosis may not be widespread, most likely as a result of inadequate resources in several countries on the continent, especially in rural and peri-urban areas. Machine learning, alongside signal processing methods and mobile communication strategies, is often used to automate the diagnosis of communicable diseases. Screening involving the use of AI has been demonstrated to be beneficial in enhancing adherence to the management of non-communicable diseases such as diabetic retinopathy.

It is also concluded that Algeria, Egypt, South Africa, and Morocco were the leading African countries in terms of published data, where the use of AI in the diagnosis of non-communicable and non-communicable diseases, such as cardiovascular, diabetes and cancer, as well as HIV/AIDS, have been extensively researched. In relation to malaria and tuberculosis, data revealed that South Africa, Nigeria, Ghana, and Morocco were the dominant countries in the integration of AI to the diagnosis, management, and adherence to medication of the designated diseases. However, there is still room for improvement for acquiring enough funding. as it is flagged as a major research outputs drawback among African countries.
